# Consumption habits of pregnant women in the Jazan region, Saudi Arabia: a descriptive study

**DOI:** 10.1186/s13104-018-3921-5

**Published:** 2018-11-16

**Authors:** Tariq Al Bahhawi, Abrar Anwar Doweri, Rawan Mohammed Sawadi, Mariam Yahya Awaji, Mada Mohammad Jarad, Zahra Yahya Sulays, Khadijah Abdulrhman Madkor

**Affiliations:** 1 Department of Family and Community Medicine, Faculty of Medicine, Jazan University, Jazan, Kingdom of Saudi Arabia; 2 Faculty of Medicine, Jazan University, Jazan, Kingdom of Saudi Arabia

**Keywords:** Pregnancy, Diet, Beverages, Medication, Jazan

## Abstract

**Objective:**

Maternal nutritional habits are critical for the health of both mother and offspring. Postpartum outcomes for mother and infant are strongly influenced by the mother’s nutritional status. Information about consumption habits among pregnant women in Saudi Arabia is scarce. Thus, this study aims to describe the consumption habits of pregnant women in the Jazan region, Saudi Arabia.

**Results:**

Meat, fish, and fruits were consumed by 97%, 86%, and 90% of the sample. Sugary desserts, fast food, and canned food were consumed by 90%, 81%, and 71% of the sample. Caffeine, juices, and milk were consumed by 75%, 92%, and 81% of the sample. Previous percentages show general higher consumption habits of food and beverages. Over-the-counter medication was used by only 17%. Folic acid, iron, and calcium use by 77%, 64%, and 58% of the sample, respectively. These percentage shows conservative use of Over-the-counter medication and sub-optimal use of important dietary supplements. Moreover, there was a positive association between caffeine intake and trimesters. Furthermore, there was negative association between education level and fish intake. Finally, canned foods consumption was higher among low income pregnant women.

## Introduction

Consumption habits during pregnancy are important from public health prospective for promotion of healthy pregnancy. These consumption habits can be either related to foods, beverages or medications. Good maternal nutrition status is crucial for women’s health and the health, survival, and development of their infants [1, 2]. The mother’s consumption habits during pregnancy are an important determent of postpartum outcomes for both mother and child [3, 4].

Some food and beverages are rich sources of essential nutrients such as vegetables, fruits, meat, sea food and milk products [5]. On the other hands, some foods and beverages contain particular components or toxins that can lead to serious consequences [6–8]. Moreover, pregnant women are advised to increase some nutrients, such as protein, carbohydrates, fat, and some vitamins and minerals in order to meet the progressive demands of physiological changes in maternal tissues and fetal growth [2, 6]. Thus, the data about food groups and beverages consumption during pregnancy are important to evaluate the pattern of foods and beverages consumption habits and approximate the healthy diet habits.

Medications are one of the most common consumed substance during pregnancy. Multinational study in Europe reported that around 80% of pregnant women used at least one medication during pregnancy (prescribed or over-the counter OTC) [9]. In the USA, around 50% of the pregnant women report use over-the counter (OTC) medications (at least one medication during pregnancy) [10]. Although, no randomized controlled trials have been conducted to produced clear evidence or guidance on the use of OTC medications during pregnancy [11].

In Saudi Arabia, a national survey revealed that only a small percentage of the Saudi population met the dietary guidelines [12]. Moreover, a study conducted among pregnant women showed the same results [13]. However, around 40% of pregnant women in Saudi Arabia use medication during pregnancy [14]. There is limited data, however, for consumption habits by pregnant women in Saudi Arabia, especially consumption of beverages and OTC medications. Therefore, this study aims to evaluate the consumption habits of pregnant women regarding food, beverages, and medications.

## Main text

### Methods

A cross-sectional study was conducted in the Jazan region in five primary health care (PHC). centers in April and May 2017. Jazan is one of the smallest regions in Saudi Arabia. The region is situated in the southwest of the country and aligns with the southern seaboard of Yemen. According to the 2015 census conducted by the Saudi General Authority of Statistics, the total population of the region is about 1.5 million.

100 pregnant women were recruited from five PHC centers chosen randomly from all Jazan PHC centers. The original sample size was calculated to be 400 pregnant women, but due to the very low number of pregnant women attending prenatal care clinics, we ended up with 76 participants. After discussion with the ethical committee, we decided to extract phone numbers from the PHC files and contacted potential participants until we reached 100.

The data were collected using a self-administered questionnaire comprising two parts. The first part contained questions about demographic information (such as age, education level, living standard, place of residence and gestational age). and the second part was the Food, Beverage, and Medication Intake Questionnaire (FBMIQ). which has been used previously [6]. The FBMIQ was originally designed to evaluate maternal consumption habits about certain foods, beverages and medications during pregnancy; it is not a comprehensive assessment of diet but rather a short survey about particular items consumed. It is assess how often and during which time are certain foods, beverages and medications consumed during the pregnancy period. On the other hand, it does not assess the portion size or number of servings. The FBMIQ was translated into Arabic and modified to fit cultural factors.

Data were entered, cleaned, and analyzed using SPSS version 20 (SPSS Inc, Chicago, IL, USA). Data analysis involved descriptive statistics and several inferential statistics techniques. The normality test for the study variables was assessed and all variables were normally distributed. The sociodemographic data were presented by frequency and percentage, except for age, which was shown by mean (M) and standard deviation (*SD*). Food, beverages, and medication data were presented by frequency and percentage. Pearson correlation coefficients between age and consumption habits were calculated. The Chi squared test and Fisher exact test were performed to determine the differences in consumption frequencies for meat, fish, fruit, canned food, desserts, fast food, milk, juice, caffeine, OTC and prescribed medication with grouping variables of level of education, income and trimesters. A p-value less than 0.05 was used as the cut-off level for statistical significance.

### Results

The questionnaires were completed by 100 pregnant women. Their demographic characteristics are summarized as the following: The sample mean age was 27.8 years, with a SD of 5.9 years and a range of 16–42 years. Of the sample, 55% had received a university degree or higher. About 42% of the sample were pregnant women in their third trimester, 40% in their second trimester, and 18% in their first trimester. Around 52% of the sample have monthly income higher than 6000 Saudi Riyal (SR) and 48% have 6000 SR or less.

Food consumption habits are presented in Table 1. The most commonly consumed category was any meat products (consumed by 97% of the pregnant women), followed by fruit and sugary desserts (90%), then fish, fast food, and canned food (86%, 81%, and 71%, respectively).

**Table 1:** Food, Beverage and medications consumption habits among pregnant women

Substance consumed n = 100	Pregnant women N (%)
Food	
Any meat	97 (97)
Meat	12 (12.4)
Chicken	9 (9.3)
Both	75 (77.3)
Other	1 (1.03)
Any fish	86 (86)
Tuna	18 (20.9)
Greasy grouper	27 (31.4)
Both	40 (46.5)
Other fish	1 (1.2)
Fresh fruit	90 (90)
Bananas	72 (80)
Oranges	78 (86.6)
Apples	74 (82.2)
Other fresh fruit	23 (25.5)
Any canned foods	71 (71)
Canned fruits/veggies	37 (52.1)
Canned soup	34 (47.9)
Canned tuna	45 (63.4)
Other canned foods	2 (2.8)
Sugary desserts	90 (90)
Ice cream	68 (75.5)
Baked desserts	65 (72.2)
Chocolate	78 (86.6)
Other desserts	2 (2.2)
Fast foods	81 (81)
Burgers	58 (71.6)
French fries	71 (87.6)
Chicken products	41 (50.6)
Other fast foods	3 (3.7)
Beverage	
Any water	100 (100)
Tap water	10 (10)
Bottled water	92 (92)
Home-filtered water	5 (5)
Other water	0 (0)
Any milk	81 (81)
Organic	1 (1.2)
Full fat	60 (74)
Low fat	19 (23.5)
Fat-free	5 (6.2)
Any juice	92 (92)
Orange juice	75 (81.5)
Apple juice	46 (50)
Mixed juice	56 (60.8)
Other juice	14 (15.2)
Caffeine	75 (75)
Coffee	51 (68)
Tea	56 (74.6)
Caffeinated drinks	45 (60)
Energy drinks	7 (7)
Red Bull	0 (0)
Code Red	4 (57.1)
Power Horse	1 (14.3)
Other	3 (42.8)
Non-alcoholic beer	5 (5)
Medication	
Over-the-counter	17 (17)
Medication	2 (11.7)
Decongestants	3 (17.6)
Cough/cold medications	0 (0)
Ibuprofen	1 (5.9)
Aspirin	14 (82)
Acetaminophen	4 (23.5)
Prescribed medication	95 (95)
Vitamins	46 (48.4)
Calcium	58 (61.05)
Morning sickness	27 (28.4)
Medications	1 (1.05)
Antidepressants	23 (24.2)
Pain medications	64 (67.4)
Iron	77 (81.05)
Folic acid	18 (18.9)

Table 1 shows the beverage consumption habits of the sample, with 100% of the women drinking water and 92% of them drinking bottled water. Juices were consumed by 92% of the respondents. Milk was consumed by 81%, followed by caffeinated beverages (75%). Finally, energy drinks and non-alcoholic beer were the beverages least-often consumed by pregnant women (7% and 5%, respectively).

Medication and vitamin consumption during pregnancy are shown in Table 1, which shows that prescribed medication was consumed by 95% of the respondents. Folic acid, iron, and calcium were the most-consumed medications, consumed by 77%, 64%, and 58%, respectively, of the respondents. OTC medications were consumed by 17% of the pregnant women.

Table 2 highlights the frequency of the consumption habits during pregnancy. Meat products were consumed 1–3 times per week for around 39% of the respondents, while fish was consumed 1–3 times per week by 50%. Fast food, fruits, sugary desserts, and canned food were consumed 1–3 times per week by 31% to 44% during pregnancy. Water was consumed on a daily basis. Milk, juice, and caffeine were consumed 1–3 times per week by 10% to 37% during pregnancy. Around 77% of OTC medications were consumed 1–3 times per month, while prescribed medications were consumed on a daily basis by 74% of respondents.

**Table 2:** Frequency of consumption habits among pregnant women

Substance consumed	1–3/entire pregnancy N (%)	1–3/month N (%)	1–3/week N (%)	4–6/week N (%)	7+/week N (%)
Food					
Meat	7 (7.3)	10 (10.4)	38 (39.6)	23 (24)	18 (18.8)
Fish	9 (10.5)	15 (17.4)	43 (50)	10 (11.6)	9 (10.5)
Fruit	6 (6.7)	10 (11.1)	31 (34.4)	19 (21.1)	24 (26.7)
Canned food	9 (12.7)	31 (43.7)	22 (31)	4 (5.6)	5 (7)
Sugary desserts	1 (1.1)	6 (6.7)	30 (33.3)	24 (26.7)	29 (32.2)
Fast food	7 (8.6)	23 (28.4)	36 (44.4)	9 (11.1)	6 (7.4)
Beverage					
Water	0	0	0	4 (4)	96 (96)
Milk	4 (4.9)	12 (14.8)	26 (32.1)	15 (18.5)	24 (29.6)
Juice	3 (3.3)	14 (15.2)	9 (9.8)	23 (25)	43 (46.7)
Caffeine	2 (2.7)	6 (8)	28 (37.3)	15 (20)	24 (32)
Medication					
Over-the-counter	8 (47.1)	5 (29.4)	0	3 (17.6)	1 (5.9)
Prescribed	3 (3.2)	6 (6.3)	6 (6.3)	10 (10.5)	70 (73.7)

No significant correlations were found for consumption habits with age. Table 3 examine the association between consumption habits and demographic variables. Caffeine consumption was the only outcome that showed statistical significance with trimesters (P-value = 0.01). Fish consumption was statistically significant with the education level (P-value = 0.002). women with collage degree or above were less consume fish than women with some school education level. According to the monthly income variable, canned food and milk consumption were statistically significant with P-value of 0.03 and 0.048, respectively. Women with lower income were consumed more canned food and less milk than women with higher income.

**Table 3:** Association of the consumption habits with demographic variables

Substance consumed n = 100	Trimester			Education		Income	
	1st N (%)	2nd N (%)	3rd N (%)	Some school N (%)	Collage and above N (%)	≤ 6000 N (%)	> 6000 N (%)
Food							
Any meat							
Yes	17 (94.4)	39 (97.5)	41 (97.6)	43 (95.6)	54 (98.2)	47 (97.9)	50 (96.2)
No	1 (5.6)	1 (2.5)	1 (2.4)	2 (4.4)	1 (1.8)	1 (2.1)	2 (3.8)
P-value	0.58^a^			0.58^a^		0.99	
Any fish							
Yes	14 (77.8)	36 (90)	36 (85.7)	44 (97.8)	42 (76.4)	43 (89.6)	43 (82.4)
No	4 (22.2)	4 (10)	6 (14.3)	1 (2.2)	13 (23.6)	5 (10.4)	9 (17.6)
P-value	0.46			0.002*		0.32	
Fresh fruit							
Yes	16 (88.9)	37 (92.5)	37 (88.1)	41 (91.1)	49 (89.1)	41 (85.4)	49 (94.2)
No	2 (11.1)	3 (7.5)	5 (11.9)	4 (8.9)	6 (10.9)	7 (14.6)	3 (5.8)
P-value	0.75^a^			0.99^a^		0.18^a^	
Any canned foods							
Yes	11 (61.1)	29 (72.5)	31.(73.8)	30 (66.7)	41 (74.5)	39 (81.3)	32 (61.5)
No	7 (38.9)	11 (27.5)	11 (26.2)	15 (33.3)	14 (25.5)	9 (18.7)	20 (38.5)
P-value	0.58			0.38		0.03*	
Sugary desserts							
Yes	14 (77.8)	36 (90)	40 (95.2)	41 (91.9)	49 (89.1)	45 (93.8)	45 (86.5)
No	4 (22.2)	4 (10)	2 (4.8)	4 (8.1)	6 (10.9)	3 (6.2)	7 (13.5)
P-value	0.11^a^			0.99^a^		0.32^a^	
Fast foods							
Yes	14 (77.8)	31 (77.5)	36 (85.7)	37 (82.2)	44 (80)	39 (81.3)	42 (80.8)
No	4 (22.2)	9 (22.5)	6 (14.3)	8 (17.8)	11 (20)	9 (18.7)	10 (19.2)
P-value	0.59			0.77		0.95	
Beverage							
Any milk							
Yes	13 (72.2)	31 (77.5)	37 (88.1)	36 (80)	45 (81.8)	35 (72.9)	46 (88.5)
No	5 (27.8)	9 (22.5)	5 (11.9)	9 (20)	10 (18.2)	13 (27.1)	6 (11.5)
P-value	0.27			0.81		0.048*	
Any juice							
Yes	16 (88.9)	37 (92.5)	39 (92.9)	41 (91.1)	51 (92.7)	43 (89.6)	49 (94.2)
No	2 (11.1)	3 (7.5)	3 (7.1)	4 (8.9)	4 (7.3)	5 (11.4)	3 (5.8)
P-value	0.79^a^			0.99^a^		0.47^a^	
Caffeine							
Yes	10 (55.6)	36 (90)	29 (69)	37 (82.2)	38 (69.1)	36 (75)	39 (75)
No	8 (44.4)	4 (10)	13 (31)	8 (17.8)	17 (30.9)	12 (25)	13 (25)
P-value	0.01*			0.13		1	
Medication							
OTC medication							
Yes	3 (16.7)	5 (12.5)	9 (21.4)	10 (22.2)	7 (12.7)	9 (18.8)	8 (15.4)
No	15 (83.3)	35 (87.5)	33 (78.6)	35 (77.8)	48 (87.3)	39 (81.2)	44 (84.6)
P-value	0.56			0.20		0.65	
Prescribed medication							
Yes	18 (100)	38 (95)	39 (92.9)	42 (93.3)	53 (96.4)	45 (93.8)	50 (96.2)
No	0 (0)	2 (5)	3 (7.1)	3 (6.7)	2 (3.6)	3 (6.2)	2 (3.8)
P-value	0.83^a^			0.65^a^		0.66^a^	

^*^ Statistically significant

^a^Based-on Fisher exact test

### Discussion

The aim of our study was to describe the consumption habits of pregnant women in the Jazan region. The mean age of the respondents was similar to that in a study conducted in Saudi Arabia: 27.8 years [13].

Meat consumption during pregnancy in our sample was 97%, with 82% of the respondents consuming meat at least once per week. These results were consistent with a study conducted in the USA. On the other hand, studies conducted in Ghana and Nigeria showed that only 33% and 48% of pregnant women, respectively, consumed meat at least once per week [15, 16]. This could be due to the economic differences in the populations. 86% of pregnant women reported fish consumption, with half reporting 1–3 time per week, Fish consumption habits were higher than those found in the USA study and lower than those in the Ghana study [6, 15]. This could be due to differences in cultural food habits or the geographical position of Jazan as a coastal region. Fruits were consumed by 90% of the respondents, with 94% consuming 1–3 time per week. High fruit consumption in our sample was consistent with the USA and Nigeria studies and higher than the Ghana study [6, 15, 16]. Although canned food contains toxic chemicals, such as Bisphenol A, which impacts mother and child health [8], Canned foods were consumed by 71% of pregnant women, with 43% of them consuming them 1–3 times per week or more, a finding similar to the USA study [6]. Sugary desserts and fast food were highly consumed (by 90% and 81% of respondents, respectively). Frequency of consumption of these foods was high, with 92% of respondents consuming dessert at least once a week, while fast food was consumed by 63% once a week. The USA study reported high consumption habits for sugary desserts and fast food but with much lower frequency than our results [6]. Maternal consumption of fast food is associated with high infant birth weight and increased asthmatic symptoms in children [17, 18]. Sugary desserts can lead to gestational diabetes and may increase the risk of ovarian cancer [6, 19].

Tap water consumption in our sample was 10%. The fact that tap water is more prone to contamination supports the idea that public water can increase the risk of preterm birth, spontaneous abortion, and low birth weight [20]. Milk and juices were highly consumed by pregnant women in our sample. Milk consumption during pregnancy can increase gestational, placental, fetal, and birth weight [21]. However, milk is a rich source of nutrients especially calcium [5]. Fresh fruits and juices that contained folic acid, such as orange and orange juice, are recommended to be consumed by pregnant women [22]. Caffeine was consumed by 75% of respondents, with 90% of them consuming caffeine at least once per week. There are inconclusive results about the acceptable level of caffeine intake by pregnant women, but recommendations propose that caffeine intake be reduced during pregnancy [23].

Over-the-counter medication use was low among pregnant women. These results was consistent with study conducted in Riyadh [14]. In contrast, another study conducted in Riyadh tertiary care hospital showed high use of OTC during pregnancy [24]. Moreover, important medical supplements use was sup-optimal for folic acid, iron, and calcium were the most consumed supplements, consumed by 77%, 64%, and 58% of the pregnant women, respectively. Folic acid use among pregnant women in Sudan was 92%, while in the USA it was 77% [25, 26]. In Saudi Arabia, study conducted in Riyadh showed a high use of dietary supplements with 96% for folic acid, iron 89% and calcium 82% [27].

Our results showed a significant positive increase in the caffeine consumption by trimesters. These results were consistent with previous finding [28]. In contrast, some studies showed no difference in caffeine consumption regard trimesters or negative association [6, 29]. These differences could be due to differences in the measurement of caffeine. Moreover, our study revealed lower consumption of fish by women with higher education level than women with lower education level. On the other hand, studies showed positive increase of fish consumption with education level [30, 31]. These differences could be due small sample size in our study. Furthermore, women with lower income consume more canned food than women with higher income. This result was reported by USA study [6]. Milk consumption was higher among high income women than low income women. In contrast, there was no significant association between milk consumption and level of income among pregnant women in the USA study [6].

In conclusion, this study summaries the consumption habits of Saudi women, revealing high consumption habits of food and beverages among pregnant women. In contrast, lower use of OTC and prescribed medication with sup-optimal intake of folic acid, iron, calcium, and vitamins during pregnancy. Moreover, there was a positive association between caffeine intake and trimesters. Furthermore, there was negative association between education level and fish intake. Finally, canned foods consumption was higher among low income pregnant women.

## Limitations

This study describes the consumption habits of pregnant women in the Jazan region. One of limitations of the study is self-reported quality, which can increase reporting bias. Moreover, the small sample size due to low prenatal care follow-up can lead to sampling bias. In addition, the questionnaire is not for complete diary assessment.

## Abbreviations

OTC: over-the counter
PHC: primary health care
FBMIQ: Food, Beverage, and Medication Intake Questionnaire
SR: Saudi Riyal
